# Artificial Light at Night Alleviates the Negative Effect of Pb on Freshwater Ecosystems

**DOI:** 10.3390/ijms20061343

**Published:** 2019-03-17

**Authors:** Gaozhong Pu, Danjuan Zeng, Ling Mo, Jianxiong Liao, Xiaxia Chen

**Affiliations:** 1 Guangxi Key Laboratory of Plant Conservation and Restoration Ecology in Karst Terrain, Guangxi Institute of Botany, Guangxi Zhuang Autonomous Region and Chinese Academy of Sciences, Guilin 541006, China; djzeng221@163.com (D.Z.); ml@gxib.cn (L.M.); njandgl@163.com (J.L.); chenxx7276@163.com (X.C.); 2 College of Life Science, Guangxi Normal University, Guilin 541006, China

**Keywords:** litter decomposition, light pollution, fungal community, metagenomics, extracellular hydrolytic enzymes

## Abstract

Artificial light at night (ALAN) is an increasing phenomenon worldwide that can cause a series of biological and ecological effects, yet little is known about its potential interaction with other stressors in aquatic ecosystems. Here, we tested whether the impact of lead (Pb) on litter decomposition was altered by ALAN exposure using an indoor microcosm experiment. The results showed that ALAN exposure alone significantly increased leaf litter decomposition, decreased the lignin content of leaf litter, and altered fungal community composition and structure. The decomposition rate was 51% higher in Pb with ALAN exposure treatments than in Pb without ALAN treatments, resulting in increased microbial biomass, β-glucosidase (β-G) activity, and the enhanced correlation between β-G and litter decomposition rate. These results indicate that the negative effect of Pb on leaf litter decomposition in aquatic ecosystems may be alleviated by ALAN. In addition, ALAN exposure also alters the correlation among fungi associated with leaf litter decomposition. In summary, this study expands our understanding of Pb toxicity on litter decomposition in freshwater ecosystems and highlights the importance of considering ALAN when assessing environmental metal pollutions.

## 1. Introduction

With the continued global growth of anthropogenic activities, there are emerging pollutants (e.g., artificial light at night/ALAN, engineered nanoparticles and pharmaceuticals, and personal care products) [1,2,3]. These emerging pollutants not only directly affect organisms and ecosystems, but also interact and synergize with other environmental pressures (such as heavy metal pollution), leading to more complex effects [4].

Among emerging pollutants, ALAN pollution, which has nearly doubled in the last two decades, has received considerable attention [3]. The negative effects of ALAN range from molecular to entire ecosystems, and modify the abundance of species, distribution and behavior [5,6,7,8,9,10,11]. Humans have historically been concentrated near freshwater environments, which may lead to freshwater ecosystems being particularly susceptible to changing light regimes at night [11]. Studies on the effects of ALAN in aquatic systems have shown that ALAN not only affects the physiological and behavioral responses of individual species and predation [3,12,13], but also alters community compositions of periphyton and sediment microbes, with implications for ecosystem level functions [5,14,15]. Although studies in this field have addressed the negative effects of ALAN on different species or communities, there is a lack of research on the effects of ALAN on some important ecological processes in the aquatic ecosystem, such as plant litter decomposition, and associated microbial community activities. The decomposition of plant litter is not only an input of nutrients into rivers, but also the supporter of many ecological processes, and can be used as an assessment of the health of river ecosystems [16,17]. However, little is known about the effect of ALAN alone or its synergy with metal pollution on plant litter decomposition and associated fungal communities. They are sensitive to contaminants and can provide a suitable model to assess contaminant effects on complex freshwater ecological systems [1,17].

Like other environmental pressures, such as temperature, oxygen depletion, and drought; excessive light will significantly change the toxicity of nanoparticles, and some heavy metals [4,18,19]. Among the heavy metals, lead (Pb) is the second most harmful pollutant, after arsenic, and can cause numerous environmental issues even at very low concentrations [20]. Many studies have demonstrated that Pb is toxic to plants [21], animals [22,23], and heterotrophic biofilms [24]. In general, eco–toxicity of Pb mostly depends on Pb chemical speciation [25], while several environmental factors, such as temperature, pH, PO_4_^3−^ and dissolved organic matter (DOM), have been proven to affect lead toxicity [25,26,27,28,29,30,31]. Meanwhile, its interactions with iron (Fe) and manganese (Mn) are all additive [32,33], and the presence of some microorganisms tends to weaken lead toxicity [34] or improve Pb uptake in plants [35]. However, its potential interactions with light exposure and associated mixture effects are largely unknown. A recent study has indicated that leaf–litter decomposition is related to sediment Pb and benthic macroinvertebrate abundance [23]. Unfortunately, these studies have not paid attention to the microorganisms associated with plant litter decomposition in freshwater systems, despite their key role in assessing ecosystem health [17]. Furthermore, interactions between a natural stressor and a toxicant can sometimes lead to a greater impact than that expected from separate pressure types [4]. For example, interactions between temperature and silver nanoparticles [36], or the effects of visible light and nano-ZnO [18,19] on leaf litter decomposition, are more pronounced than expected from either of the stress types alone.

Hence, in the present study we evaluated the effect of ALAN and/or Pb on the process of leaf litter decomposition in freshwater ecosystems. For this purpose, we conducted an aquatic microcosm experiment on *Pterocarya stenoptera* leaf litter decomposition to evaluate whether ALAN altered the effect of Pb on the decomposition of the leaf litter and the associated fungal community structure, metabolic activities, and microbial biomass.

## 2. Results

### 2.1. Water Chemistry

The chemical characteristics of stream waters in microcosms were significantly different among treatments at the end of the experiment (Table 1). For example, compared with the control (N treatment), the ALAN treatment resulted in significant decreases in total suspended solids (TSS); the N-Pb and A-Pb treatments showed significant decreases in turbidity (NTU) and TSS, but significant increases in conductivity. The A-Pb treatment had significant decreases in NH_4_^+^ concentration and NTU.

### 2.2. Leaf Litter Chemical Characteristics and Decomposition Rates

**Figure 1:**
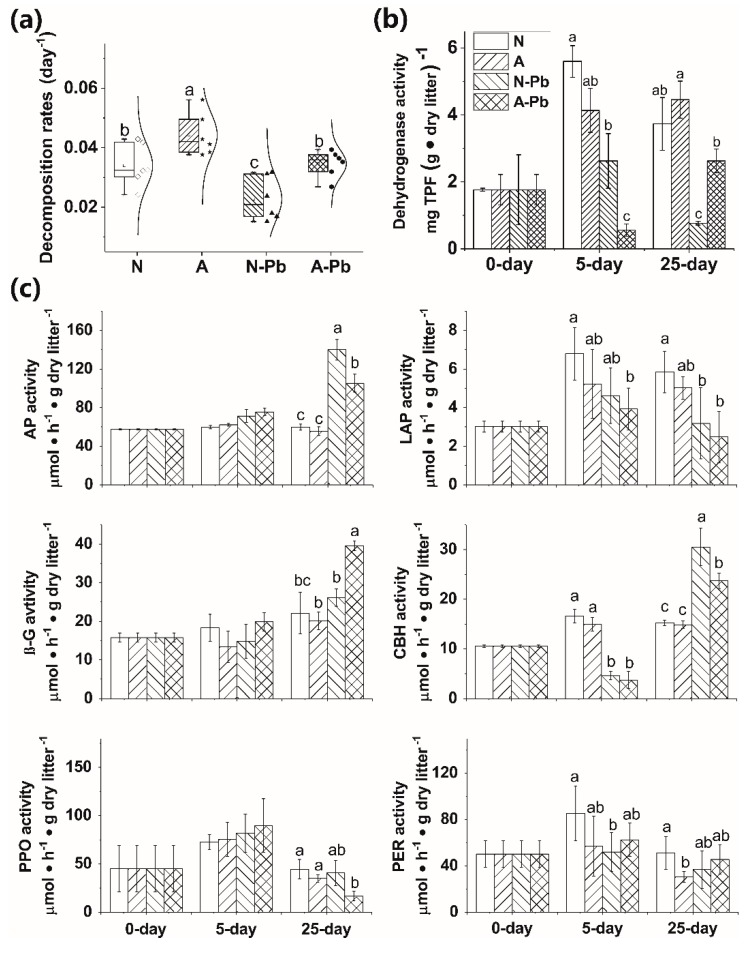
Decomposition rates of *Pterocarya stenoptera* leaf litter (**a**), associated dehydrogenase (**b**), and extracellular enzyme activities (**c**), after 25 days of incubation in the microcosms. Legend: N, natural light simulation group without Pb; A, artificial light at night (ALAN) simulation group without Pb; N-Pb, natural light simulation group with Pb; N-As, ALAN simulation group with Pb; AP, acid phosphatase; LAP, leucine-aminopeptidase; β-G, β-glucosidase; CBH, cellobiohydrolase; PPO, polyphenol oxidase; PER, phenol oxidase. Different lowercase letters on top of the bars denote significant differences (*p* < 0.05) among the treatments.

ALAN exposure alone, or combined with Pb, significantly decreased lignin content compared with the other treatments (Table 2). The ALAN only exposure significantly increased the leaf litter decomposition of *P. stenoptera* compared with the other treatments (Figure 1a). Meanwhile, the decomposition rate was 51% higher in the A-Pb treatment than in the N-Pb treatment and showed a significant difference (*p* < 0.05).

### 2.3. Microbial Biomass

The microbial activities, determined by dehydrogenase activity (DHA), were affected by both the treatments and exposure times (*p* < 0.01, Tukey’s test). The Pb only exposure, or combined with ALAN, significantly decreased the activity of DHA compared with the N treatment (with an exception of the N-Pb treatment after day 25) (Figure 1b). It is worth noting that A-Pb exposure significantly decreased the activity of DHA compared with the N-Pb treatment after 5 days, but the opposite situation occurred after 25 days (Figure 1b). 

### 2.4. Changes in Extracellular Enzyme Activities

**Figure A1:**
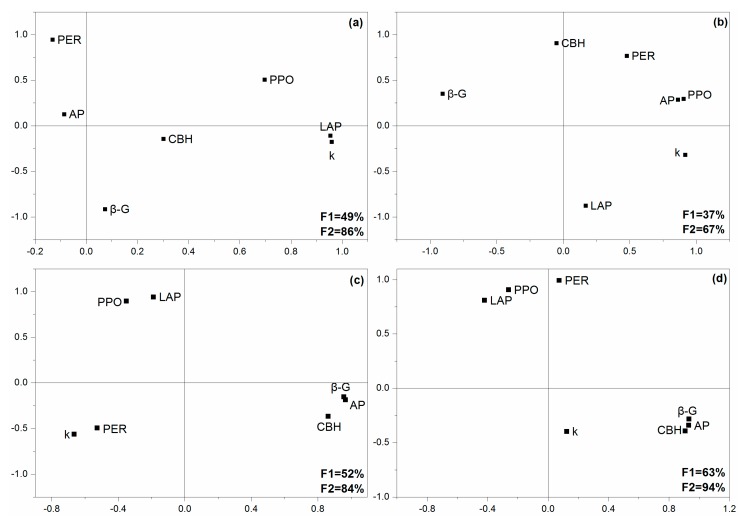
Principal component analysis of the correlation patterns of enzymatic activities and litter decomposition rates (k) in different treatments: (**a**) Natural light simulation group without Pb (N); (**b**) ALAN simulation group without Pb (A); (**c**) natural light simulation group with Pb (N-Pb); and (**d**) ALAN simulation group with Pb (A-Pb). Legends: AP, acid phosphatase; LAP, leucine-aminopeptidase; β-G, β-glucosidase; CBH, cellobiohydrolase; PPO, polyphenol oxidase; PER, phenol oxidase.

The extracellular enzyme activities (EEA) significantly varied with treatments and exposure times, except for polyphenol oxidase (PPO) and phenol oxidase (PER) (Table A1). After 5 days, the activities of leucine-aminopeptidase (LAP) and cellobiohydrolase (CBH) were significantly decreased when exposed to Pb (Figure 1c). After 25 days, the activities of acid phosphatase (AP) and CBH in both N-Pb and A-Pb treatments, as well as β-glucosidase (β-G) in the A-Pb treatment, were significantly higher than those in the control (N treatment) (Figure 1c). However, the activities of LAP in both N-Pb and A-Pb treatments, and PPO in the A-Pb treatment, were significantly higher than in the control (N treatment) (Figure 1c). Compared with the control (N treatment), ALAN decreased the correlations between LAP, β-G, and the litter decomposition rate but enhanced the correlations between AP, PER, PPO, and the litter decomposition rate (Figure A1a,b). Compared with N-Pb, A-Pb treatment increased the correlations between β-G, CBH, AP, and the litter decomposition rate (Figure A1c,d).

### 2.5. Fungal Communities

**Figure 2:**
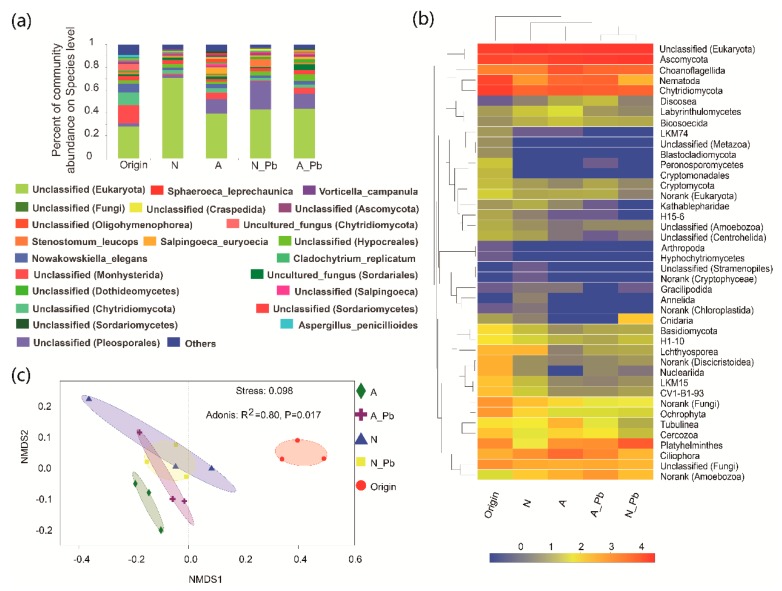
Changes in fungal taxonomic compositions at the species (**a**), and phylum (**b**) levels, and fungal community analysis by nonmetric multidimensional scaling (**c**), before and after exposure to ALAN and/or Pb. The color scale indicates the relative abundance of fungal phylum in log_2_ scale, with red representing a high relative abundance and blue, a low relative abundance. Treatment abbreviations are defined in Figure 1.

**Figure 3:**
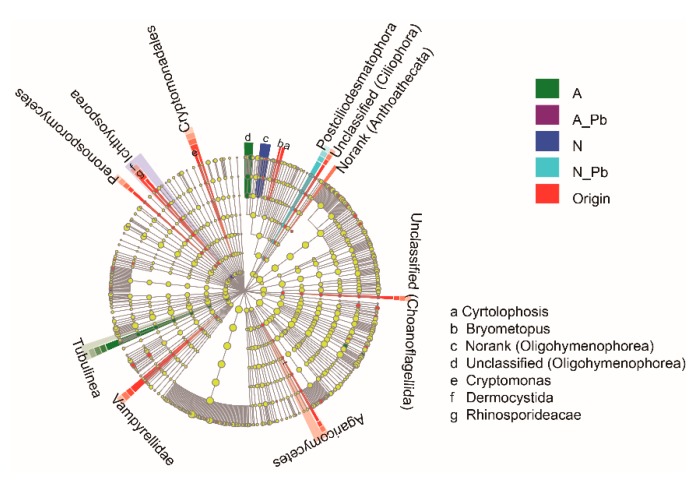
Linear discriminant analysis effect size (LEfSe) cladogram of fungal communities before and after exposure to ALAN and/or Pb. Treatment abbreviations are defined in Figure 1.

There were significant differences in the number of sequences between control (N), ALAN, and both Pb treatments, as well as in the number of operational taxonomic units (OTUs) between control (N) and other treatments (Table 3). At the species level, the abundance of the six most abundant species greatly varied in origin and different treatments (Figure 2a). For example, the relative abundance of a new species found in the present study named unclassified_d__Eukaryota was 28.07%, 70.81%, 39.74%, 43.01%, and 43.74% in the origin, N treatment, ALAN treatment, N-Pb treatment, and A-Pb treatment samples, respectively (Figure 2a). Notably, ALAN exposure significantly increased the abundance of Monhysterida, and the presence of ALAN and/or Pb significantly increased the abundance of Pleosporales. At the phylum level, unclassified phyla of Ascomycota and Chytridiomycota were the most abundant groups in all samples, but the second most abundant groups, including Nematoda, Choanoflagellida and Platyhelminthes, varied between different treatments (Figure 2b). The heat map also shows that the compositions of dominant fungal phyla in both Pb treatments were more similar to each other, and different from those of the origin, control (N treatment) and ALAN treatments (Figure 2b). Non-metric multidimensional scaling (NMDS) analysis further confirmed that the fungal communities from ALAN treatments were clearly separated from other treatments along the NMDS2 axis (Figure 2c). To better visualize and explore the data, linear discriminant analysis effect size (LEfSe) was performed (Figure 3). LEfSe analysis showed that each treatment (except the A-Pb treatment) had its own fungal indicator taxa, from class to genus level. For example, *Agaricomycetes*, *Vampyrellidae, Peronosporomycetes, Cryptomonadales, Dermocystida, Cyrtolophosis* and *Bryometopus* were enriched in the original samples; *Ichthyosporea* and an unclassified genera (Oligohymenophorea) were enriched in the N treatment; *Tubulinea* and an unclassified genera (Oligohymenophorea) were enriched in the ALAN treatment; *Postciliodesmatophora* was enriched in the N-Pb treatment. 

**Figure 4:**
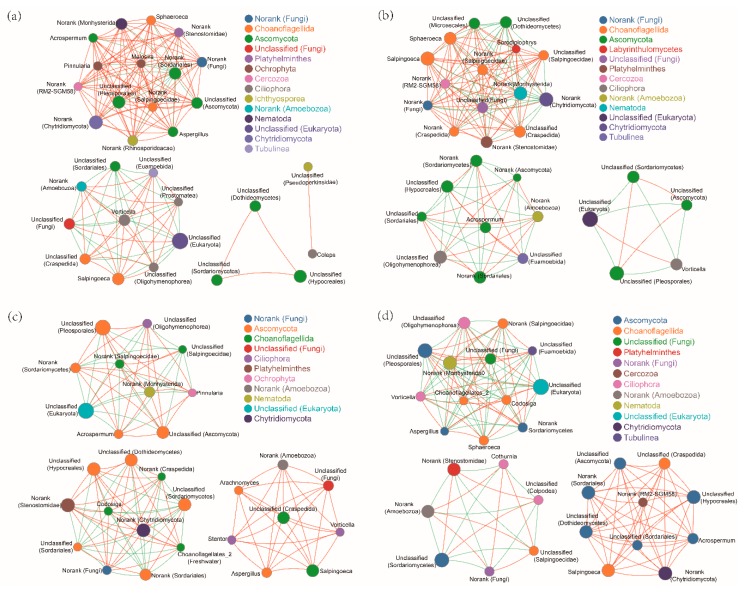
An overview of fungal networks at the genus level in different treatment groups: (**a**) natural light simulation group without Pb (N); (**b**) ALAN simulation group without Pb (A); (**c**) natural light simulation group with Pb (N-Pb); and (**d**) ALAN simulation group with Pb (A-Pb). Sizes and colors of the nodes represent the relative abundance of the fungi. Solid lines in red and green denote positive and negative correlations, respectively. The width reflects the strength of the correlation.

Correlation network analysis also showed that, compared with N treatment, ALAN and/or Pb treatments, all changed the fungal cluster group (Figure 4). Although there were three similar fungal clusters in both Pb treatments, both the genera types and correlation degrees were different (Figure 4c,d). For example, there were eight and 10 genera that were only positively correlated in the N-Pb and A-Pb treatments, respectively. Among these positively correlated genera, 60% of the genera in the A-Pb treatment belonged to the Ascomycota family (Figure 4d).

## 3. Discussion

The importance of assessing ecosystem health by plant litter decomposition in freshwater is gaining attention due to associated microbial communities and activities [1,17,18,19,36]. Here, we evaluated whether the impact of heavy metals (specifically, Pb) on freshwater ecosystems was affected by an emerging pollutant, ALAN. We show that ALAN significantly affects the toxicity of Pb on the plant litter decomposition process. This highlights the importance of considering emerging pollutants when examining heavy metal toxicity in freshwater ecosystems.

Previous studies have indicated that light (solar radiation) plays a dual role in plant litter decomposition in terrestrial ecosystems, in which solar radiation can increase plant litter decomposition via both photochemical mineralization and microbial facilitation, or negatively impact microbial growth [37,38,39]. Our results showed that ALAN increased leaf litter decomposition and decreased lignin content, indicating that ALAN exposure can stimulate litter decomposition directly by increasing the microbial accessibility to lignin, as well as by increasing the labile carbon supply to microbes since lignin, as an effective light-absorbing compound, can preferentially degrade over a wide range of wavelengths [37,39]. In addition, variation in the fungal community structure is also an important factor affecting leaf litter decomposition [19,40,41]. Our results showed that the relative abundance of the most abundant species (named unclassified_d__Eukaryota) was significantly lower in ALAN treatments than that in the control (N treatment) but the next most abundant species, including ones named unclassified_o__Monhysterida and unclassified_o__Pleosporales, were significantly higher in ALAN treatments (Figure 2a). LEfSe analysis indicated that the fungal communities of ALAN treatment, possessing their own fungal cluster group and indicator taxa from the phylum to genus level (Figure 3), were clearly separated from those of the other treatments (Figure 2c). These results indicated that ALAN can alter the fungal community composition and structure associated with leaf litter decomposition in aquatic ecosystems. Previous studies have also shown that, in aquatic ecosystems, light (UV radiation or ALAN) can not only accelerate the breakdown of complex aromatic compounds [42], but also alter the community composition of riparian predators and scavengers [43], as well as benthic primary producers [15] and sediment microbial communities [11]. Some recent studies have shown that 24 h light exposure increases the relative abundance of species *Dendrospora tenella* and genera *Massarina* and *Lunulospora* but decreases the abundance of *Dactylaria longidentata* associated with the leaf litter decomposition of *Typha angustifolia* or *Populus nigra* [18,19]. However, we did not find these fungal species or genera in the present study. One reason may be the different leaf litter types, which differ in carbon, nitrogen, and lignin content. Another reason may be the different methods of fungal assay. Du et al. [18,19] estimated fungal biodiversity by counting the fungal spores released from leaf disks, which would have missed a large amount of unculturable fungal data. In the present study, the microbial community structure was assessed by MiSeq sequencing, which can obtain both unculturable and culturable fungi (an average of 177 species), indicating that MiSeq sequencing is more appropriate for monitoring microbial communities associated with litter decomposition in aquatic ecosystems. Elevated litter decomposition rates, decline of lignin, and changes in fungal compositions indicated that ALAN can alter plant litter decomposition via both photodegradation of lignin, and the variation of fungal communities associated with leaf litter decomposition.

Another important insight from our experiments is that the negative effect of Pb on the leaf litter decomposition of *P. stenoptera* was alleviated by ALAN. In this study, we found that the decomposition rate was 51% higher in the A-Pb treatment than in the N-Pb treatment. The main reason may be the decreased lignin content of the leaf litter in the A-Pb treatment compared with the N-Pb treatment, since light exposure can increase litter biodegradability by increasing the microbial accessibility to lignin [35]. The other reason may contribute to the increased EEA associated litter decomposition. Our result showed that ALAN exposure not only increased the activity of β-G but also enhanced the relationship between β-G and litter decomposition rates after 25 days, indicating that β-G may play an important role in regulating the inhibition of leaf litter decomposition under ALAN exposure, since β-G is involved in the degradation of polysaccharide compounds [1]. However, Du et al. [18,19] found that light exposure has no effect on litter decomposition under nano-ZnO pollution. The possible reason may be the different leaf litter types, pollutants, and exposure times. In addition, the present study only conducted indoor simulated experiments with short-term exposures to ALAN. Future studies should assess the long-term effects of ALAN in the field, since field experiments can provide more accurate results.

## 4. Materials and Methods

### 4.1. Preparation of P. stenoptera Leaf Litter

The leaves of *P. stenoptera* were collected just after abscission on 15, September 2017 at Lijiang River (25°51′13.5″N, 110°24′59.1″E, altitude of 433 m, southeastern lowlands of China) and dried at room temperature. Leaf discs were cut out with a 14-mm diameter cork borer and sets of 100 leaf disks were enclosed in fine mesh bags (18 × 25 cm, 0.5-mm mesh size). In December 2017, 30 fine mesh bags were immersed in a first-order hardwater stream of the Lijiang River to allow fungal colonization. Three litter bags were sampled after 30 min to determine the initial mass of leaf disks. After 14 days, all the littler bags were collected, placed into a cool box with stream water, and then returned to the laboratory. During leaf immersion, stream water characteristics were measured in situ using a SEBA MPS-Checker (MPS572, Kaufbeuren, Germany) and the results are shown in Table A2. In addition, 50 L stream water was collected, returned to the laboratory and stored at −20 °C until use.

### 4.2. Experimental Design

After retrieval from the stream, three litter bags were used for original data analysis including the original fungi biodiversity, EEA, and microbe biomass evaluation. The other litter bags were rinsed with deionized water and sets of 100 leaf discs were randomly distributed into sterile Erlenmeyer flasks (150 mL) with 80 mL of sterile stream water (six flasks for each of the four experimental conditions), which was supplemented with Pb (C_4_H_6_O_4_Pb·3H_2_O) at two concentrations (0 and 1000 μg L^−1^) under natural light simulation (N group, with a natural light:dark ratio of 12 h:12 h) or ALAN simulation (A group, with a natural light:artificial light ratio of 12 h:12 h). In each group, there were two different treatments: Without Pb addition (N or A) and with Pb addition (N-Pb or A-Pb, 1000 μg L^−1^). To maximize the natural light conditions in both natural light and ALAN simulation groups, the shakers with glass observation ports were placed in a greenhouse with a roof and walls made of glass. To simulate ALAN conditions (at night), four small LED lights (30 W, NVC 6500 K, Zhejiang, China) with an output spectrum ranging from 400 nm to 750 nm were used in the shaker, and all glass observation ports of the shaker were screened with awning cloth only at night. Meanwhile, the natural light simulation group was only exposed to natural light at night. In the ALAN simulation group, the mean illumination was 180 ± 13 lux at night, while in the natural light simulation group, it was 0.11 ± 0.03 lux at night. The mean illumination was 503 ± 24 lux during the day. Microcosms were incubated (18 °C, 150 rpm) for 25 days, and the solutions including C_4_H_6_O_4_Pb·3H_2_O were renewed every 5 days. After exposure times of 5 and 25 days, sets of 12 microcosms (four experimental treatments, replicated in triplicate) were immediately sampled to determine the leaf litter mass loss, chemical characteristics, microbial biomass, EEA and the fungal community composition.

### 4.3. Leaf Mass Loss and Chemical Characteristics

Remaining leaf mass was determined as described by Woodward et al. [17] and decomposition rates (*k*) were calculated as follows:

*x*_0_ = *x_t_ · e^−kt^*

where *x*_0_ is the initial mass and *x_t_* is the remaining mass at time *t*. 

Total nitrogen and carbon concentrations were measured using an element analyzer (Elementar Vario MACRO, Germany) after digestion with sulfuric acid and potassium sulfate at 360 °C. The lignin concentration was measured following the method of Gessner [44].

### 4.4. Microbial Biomass

Dehydrogenase activity (DHA) has been used to evaluate the overall microbial biomass, since there is significant correlation between the two [45]. DHA measurements followed Hoostal et al. [46] with slight modifications. Briefly, three leaf litter disks with 0.4 mL of triphenyltetrazolium chloride (TTC) solution (pH of 7.6) were incubated in the dark at 25 °C for 24 h. Then, 4 mL of acetone was added and incubated for 2 h in the dark at room temperature. The solution was measured at 485 nm by using a spectrophotometer (PERSEE General Instrument Inc., Beijing, China). Three autoclaved leaf discs were used as the negative controls for each sample.

### 4.5. Extracellular Enzyme Activities

Three leaf discs were used to determine the EEA according to the methods described in the Allison Lab Protocol [47]. Activities of six extracellular enzymes were determined, namely four hydrolases (β-glucosidase (β-G), cellobiohydrolase (CBH), leucine-aminopeptidase (LAP), alkaline phosphatase (AP)) and two oxidases (polyphenol oxidase (PPO) and peroxidase (PER)) that are involved in the cycling of carbon, nitrogen, phosphorous and in oxidative degradation.

### 4.6. Fungal Diversity and Community Structure

Fungal diversity and community structured associated with litter decomposition were determined using Illumina MiSeq sequencing technology. The original leaf discs (origin) at day 0 and the leaf discs sampled from the microcosms at day 25 were used to analyze the fungal community. Genomic DNA was extracted in triplicate from 0.2 g of samples using an EZNA Plant DNA Kit (OMEGA Bio-Tek, Norcross, GA, USA). The V5 region of the 18S rRNA gene was amplified using the primers SSU0817F (5′-TTAGCATGGAATAATRRAATAGGA-3′) and 1196R (5′-TCTGGACCTGGTGAGTTTCC-3′). The PCR reactions were conducted using the following program: 3 min of denaturation at 95 °C, 27 cycles of 30 s at 95 °C, 30s for annealing at 55 °C, 45 s for elongation at 72 °C, and a final extension at 72 °C for 10 min [48]. PCR reactions were performed once for each of the triplicated samples in a 20 μL mixture containing 4 μL of 5 × FastPfu Buffer, 2 μL of 2.5 mM dNTPs, 0.8 μL of each primer (5 μM), 0.4 μL of FastPfu Polymerase, and 10 ng of template DNA. The resultant PCR products were extracted from a 2% agarose gel, further purified using the AxyPrep DNA Gel Extraction Kit (Axygen Biosciences, Union City, CA, USA) and quantified using QuantiFluor™-ST (Promega, Madison, WisconsinUSA) according to the manufacturer’s protocol. High-throughput pyrosequencing was performed on the Illumina MiSeq platform (Majorbio Bio-Pharm Technology Co., Ltd., Shanghai, China). Operational taxonomic units (OTUs) were clustered with a 97% similarity cut-off using UPARSE (vision 7.1, http://www.drive5.com/uparse/). Raw FASTQ files were demultiplexed, quality filtered by Trimmomatic v0.32 [49], and merged by FLASH [50]. The taxonomy of each 18S rRNA gene sequence was analyzed with the RDP Classifier algorithm [51]. 

### 4.7. Data Analysis

Differences in the leaf litter decomposition, DHA, EEA, and fungal composition amongst treatments were tested for significance using a one-way analysis of variance (ANOVA). Principal component analysis (PCA) was performed to determine the correlation patterns of litter decomposition rates and EEA. A heatmap of the relative abundance of fungal phyla in each treatment was performed with R software (V 3.2.0, R Development Core Team, R Foundation for Statistical Computing, Vienna, Austria). Non-metric multidimensional scaling (NMDS) was performed based on Bray–Curtis distances to identify differences in fungal community compositions amongst treatments. To identify the fungal groups that were significantly different amongst treatments, a LEfSe (LDA = 2) method was used with the MASS package in R. Network analyses were performed using Networkx to gain a better understanding of the fungal interactions in different treatments.

## 5. Conclusions

In conclusion, we show that ALAN exposure significantly increased leaf litter decomposition rates, decreased the lignin content of leaf litter, altered the fungal community composition and the correlation among fungi associated with leaf litter decomposition. Notably, the negative effect of Pb on the leaf litter decomposition of *P. stenoptera* was alleviated by ALAN. These results highlight the importance of considering ALAN during the assessment of the risk posed by metals to freshwater biota and ecosystem processes. 

## Figures and Tables

**Table 1:** Hydrographical and chemical characteristics of stream waters during leaf immersion and decomposition in microcosms.

Tr	pH	DO (mg·L^−1^)	NH_4_^+^ (ppm)	NTU	TDS (mg·L^−1^)	TSS (mg·L^−1^)	Cond. (μs·cm^−1^)
Origin	6.77^c^	5.32	41^c^	434^b^	24.400^a^	2.033^a^	0.036^c^
N	7.34^ab^	4.58	57^b^	612^a^	0.011^b^	2.489^a^	0.017^c^
A	7.50^a^	4.72	77^a^	678^a^	0.012^b^	0.003^c^	0.016^c^
N-Pb	7.21^b^	4.65	64^b^	225^b^	0.072^c^	0.901^b^	0.107^b^
A-Pb	7.18^b^	5.71	20^c^	235^b^	0.074^c^	0.794^b^	0.115^a^

Note: Tr, treatments; DO, dissolved oxygen; NTU, turbidity; TDS, total dissolved solids oxygen; TSS, total suspended solids; Cond., conductivity; N, natural lighting simulation group; A, artificial light at night simulation group. Different lowercase letters denote a significant difference between treatments (*p* < 0.05), the same is true for subsequent tables.

**Table 2:** Carbon, nitrogen, phosphorus, and lignin contents presented as mg g^−1^ of dry litter mass.

Treatments	Carbon (mg·g^−1^)	Nitrogen (mg·g^−1^)	Phosphorus (mg·g^−1^)	Lignin (%)
N	286.73^b^	25.29	1.32	5.10^a^
A	348.04^ab^	26.48	1.28	2.96^c^
N-Pb	419.64^a^	27.84	1.38	6.76^a^
A-Pb	333.61^ab^	28.96	1.41	3.13^b^

Note: Treatment abbreviations are defined in Table 1.

**Table 3:** Abundance and diversity of fungal and bacterial communities associated with litter decomposition.

Samples	Sequence	OTUs	Sobs	Shannon	Simpson	ACE	Chao1
Origin	58,462^a^	351^a^	274^a^	3.08^a^	0.15^b^	293^a^	293^a^
N	49,342^b^	319^b^	219^b^	1.98 ^b^	0.38^a^	246^b^	251^b^
A	49,508^b^	275^c^	201^b^	2.60^a^	0.21^b^	219^b^	217^b^
N-Pb	57,400^a^	277^c^	202^b^	2.09^b^	0.33^a^	233^b^	234^b^
A-Pb	59,372^a^	286^c^	206^b^	2.54^a^	0.21^b^	236^b^	236^b^

Note: Treatment abbreviations are defined in Table 1.

## Appendix A

**Table A1:** Two-way ANOVA of the different treatments and sampling time on potential extracellular enzyme activities.

	AP	β-G	LAP	CBH	PPO	PER
**Treatments**						
***F***	87.54	14.83	5.95	4.96	0.04	0.53
***P***	<0.001	<0.001	0.003	0.008	0.989	0.667
**Sampling time**						
***F***	137.22	56.00	10.65	221.92	5.36	1.64
***P***	<0.001	<0.001	<0.001	<0.001	0.012	0.215
**Treatments × Sampling time**						
***F***	57.99	8.03	1.69	69.64	0.223	0.19
***P***	<0.001	<0.001	0.167	<0.001	0.955	0.976

Note: Significance (in bold) is considered at *p* < 0.05. AP, acid phosphatase; β-G, β-Glucosidase; CBH, cellobiohydrolase; LAP, leucine-aminopeptidase; PPO, polyphenol oxidase; PER, peroxidase.

**Table A2:** Hydrographical and chemical characteristics of stream waters during leaf immersion.

**Par.**	**pH**	**DO (mg·L^−1^)**	**Cond. (μs·cm^−1^)**	**Sal**	**NTU**	**OPR (mv)**	**Chla (μg·L^−1^)**	**Ca^2+^ (mg·L^−1^)**
water	6.21	3.88	0.033	0.013	0.47	154	0.65	75
**Par.**	**TSS (mg·L^−1^)**	**DOC (mg·L^−1^)**	**TP**	**NH_4_^+^ (ppm)**	**Temp. (°C)**	**TN**	**Mg^2+^ (mg·L^−1^)**	**Pb^3+^ (mg·L^−1^)**
water	3.55	1.25	0.05	0.73	20.01	1.44	4.12	< 0.01

Note: Par. = parameters; DO = dissolved oxygen; Cond = conductivity; Sal = salinity; TSS = total suspended solids; DOC = dissolved organic carbon; TN = total nitrogen; TP = total phosphorus; Chl-a = chlorophyll-a; NTU = turbidity; OPR= oxidation reduction potential; Temp. = temperature.
